# Association between maternal comorbidity and preterm birth by severity and clinical subtype: retrospective cohort study

**DOI:** 10.1186/1471-2393-11-67

**Published:** 2011-10-04

**Authors:** Nathalie Auger, Thi Uyen Nhi Le, Alison L Park, Zhong-Cheng Luo

**Affiliations:** 1 Institut national de santé publique du Québec, 190, boulevard Crémazie Est, Montréal, Québec, H2P-1E2, Canada; 2 Research Centre of the University of Montréal Hospital Centre, 3875 rue Saint-Urbain, Montréal, Québec, H2W-1V1, Canada; 3 Department of Social and Preventive Medicine, University of Montréal, C.P. 6128, succursale Centre-ville, Montréal, Québec, H3C-3J7, Canada; 4 Department of Obstetrics and Gynecology, CHU Sainte Justine, University of Montréal, 3175 Cote-Sainte-Catherine, Montréal, Canada

## Abstract

**Background:**

Preterm birth (PTB) is a major cause of infant morbidity and mortality, but the relationship between comorbidity and PTB by clinical subtype and severity of gestational age remains poorly understood. We evaluated associations between maternal comorbidities and PTB by clinical subtype and gestational age.

**Methods:**

We conducted a retrospective cohort study of 1,329,737 singleton births delivered in hospitals in the province of Québec, Canada, 1989-2006. PTB was classified by clinical subtype (medically indicated, preterm premature rupture of membranes (PPROM), spontaneous preterm labour) and gestational age (< 28, 28-31, 32-36 completed weeks). Odds ratios (OR) of PTB by clinical subtype for systemic and localized maternal comorbidities were estimated using polytomous logistic regression, adjusting for maternal age, grand multiparity, and period. Attributable fractions were calculated.

**Results:**

PTB rates were higher among mothers with comorbidity (10.9%) compared to those without comorbidity (4.7%). Several comorbidities were associated with greater odds of medically indicated PTB compared with no comorbidity, but only comorbidities localized to the reproductive system were associated with spontaneous PTB. Drug dependence and mental disorders were strongly associated with PPROM and spontaneous PTBs across all gestational ages (OR > 2.0). At the population level, several major comorbidities (placental abruption, chorioamnionitis, oliogohydramnios, structural abnormality, cervical incompetence) were key contributors to all clinical subtypes of PTB, especially at < 32 weeks. Major systemic comorbidities (preeclampsia, anemia) were key contributors to PPROM and medically indicated PTBs.

**Conclusions:**

The relationship between comorbidity and clinical subtypes of PTB depends on gestational age. Prevention of PPROM and spontaneous PTB may benefit from greater attention to preeclampsia, anemia and comorbidities localized to the reproductive system.

## Background

Preterm birth (PTB) is a major cause of mortality and morbidity throughout life and rates are increasing in many countries [1-3]. The determinants of PTB remain poorly understood [1]. Evidence suggests that maternal comorbidities are associated with PTB, especially preeclampsia, infection, uterine anomalies, and placental complications [1,4-6]. Many studies have not considered PTB by clinical subtype [5,6]. Associations with comorbidities are particularly unclear for preterm premature rupture of membranes (PPROM) and spontaneous preterm labour with intact membranes [1], the two subtypes of spontaneous PTB that are most challenging to prevent [7,8]. While medically indicated PTB has clearly been linked with ischemic placental disease [4,9], relationships remain to be established for spontaneous PTB which appears to be more closely linked with infectious processes [1,8,10,11]. However, studies are conflicting [7] and often limited by misclassification of gestational age or PTB clinical subtype [4,6,12].

Fewer studies have examined the relationships between comorbidity and PTB clinical subtype at different gestational ages, despite evidence suggesting that factors triggering PTB may differ depending on gestational age [5,13]. Research suggests that preeclampsia, uterine bleeding, cervical incompetence and chorioamnionitis may be more strongly associated with PTB at < 32 weeks of gestation compared with later PTB, though clinical subtype was not differentiated [6]. Research of PTB before 28 or 34 weeks has found associations with preeclampsia, placental abruption, chorioamnionitis, oligohydramnios, cervical insufficiency, and fetal factors [14,15], but again without evaluating clinical subtype. Information on how maternal comorbidities influence PTB rates at early gestational ages is needed, especially since PTBs below 32 weeks are responsible for nearly 50% of long term neurologic morbidity and 60% of perinatal mortality, despite accounting for only 1-2% of live births [2].

This gap in the literature needs to be addressed to better understand which maternal comorbidities to target for prevention of PTB at different gestational ages, especially PPROM and spontaneous preterm labour which are difficult to prevent [7,8]. This study sought to evaluate the associations between major maternal comorbidities and PTB by clinical subtype and gestational age.

## Methods

### Data and variables

This study was based on a retrospective cohort of all births in Québec hospitals from 1989 to 2006 (N = 1,351,211). Nearly 100% of infants in Québec are delivered in hospitals [16]. Maternal birth records were extracted from hospital discharge abstracts using the ninth revision of International Classification of Disease (ICD) codes for delivery (650, and 640-676 with 1 or 2 in the fifth position) recorded as the principal diagnosis or one of fifteen secondary diagnoses [16]. Hospital transfers (N = 21) and foreign visitors with no provincial health insurance number (N = 15,399) were excluded. We also excluded multiple births and stillbirths (646.0, 651, 652.6, 656.4, 678.1, V271-V277) and pregnancy terminations (779.6) for which mechanisms driving PTB may differ (N = 21,471).

Births at < 37 completed weeks of gestation were defined as PTBs [1]. Gestational age estimates in Québec are ultrasound-based which may be more accurate than estimates based on recall of date of last menstruation [13]. Recall-based measures are a common limitation of population-based research [12]. Clinical subtypes of PTB included medically indicated, PPROM, and spontaneous preterm labour [1,9,17-19]. Spontaneous PPROM was identified using ICD-9 codes 658.2-658.4 among births < 37 weeks. Medically indicated PTB cases that were not secondary to PPROM were identified using procedure codes for labour induction (855, 850.1, 851.9) and cesarean delivery (860-862, 868, 869) [16]. The remaining PTBs were designated spontaneous preterm labour with intact membranes, hereafter denoted spontaneous PTB. PTB subtypes were further categorized as extreme (< 28 weeks), very (28-31 weeks), and moderate (32-36 weeks) [13,17]. Four births with missing data on gestational age were excluded, leaving 1,329,737 cases in the final analysis cohort. Births at extremely early gestational ages (under 20 weeks, n = 565; 0.04%) were not excluded, as they may represent true cases of PTB [7] (and sensitivity analyses excluding these cases yielded similar findings).

Maternal comorbidities based on the ICD-9 [20] were classified in two broad categories to facilitate data classification (systemic comorbidities vs. comorbidities localized to the reproductive tract). Common comorbidities recorded as the principal or secondary diagnosis were grouped into sub-categories within these two broad categories. Systemic comorbidities representing a more generalised maternal disease process included hypertension (preeclampsia/eclampsia, pre-existing, gestational, unspecified), cardiovascular disease, diabetes (pre-existing, gestational), edema/renal disease, genitourinary infection, general infection, thyroid disease, anemia, drug dependence, mental disorders, and other comorbidity. Localized comorbidities included hemorrhage (placental abruption, placenta previa, other), chorioamnionitis, amniotic sac disorders (polyhydramnios, oligohydramnios, unspecified), cervical incompetence, structural abnormality, previous cesarean delivery, and fetal factors (anomaly, other). ICD-9 codes are provided (Additional file 1). Comorbidity variables were expressed categorically for hypertension, diabetes, hemorrhage, amniotic sac disorders, and fetal factors, and dichotomously for the remaining conditions.

Covariates included maternal age (< 20, 20-24, 25-29, 30-34, 35-39, ≥ 40 years), grand multiparity (ICD-9 659.4; ≥ 5 versus < 5 previous live births) [21], and period (1989-1993, 1994-1997, 1998-2001, 2002-2006).

### Statistical analysis

Descriptive statistics (n, %) were computed. Logistic regression was used to estimate odds ratios (OR) and 95% confidence intervals (CI) for comorbidity indicators and overall preterm birth (without considering clinical subtype) in models that were unadjusted and adjusted for maternal age, grand multiparity and period. Polytomous logistic (or multinomial) regression [22,23] was used to evaluate adjusted associations between each comorbidity and PTB subtype (medically indicated, PPROM, spontaneous) for each gestational age category in models using term births (≥ 37 weeks) as the referent [13]. Polytomous models containing one indicator of comorbidity at a time were run using the glogit option of the LOGISTIC procedure in SAS [23]. To evaluate the extent to which PTB rates were attributable to each comorbidity, population attributable fractions were computed with the formula ((RR-1)/RR)*(exposed cases/overall cases) [24,25]. Analyses were performed with SAS version 9.2 (Statistical Analysis System, Cary, North Carolina) and SPSS version 17.0 statistical software. This study conformed to the 2010 Tri-Council Policy Statement for ethical conduct of research involving humans in Canada. Approval was waived by the research ethics committee of the University of Montreal Hospital Centre.

## Results

Overall, 6.2% of births were preterm, of which 28.7% were medically indicated, 30.7% PPROM, and 40.6% spontaneous. Over 5% of PTBs occurred at < 28 weeks, 7.3% at 28-31 weeks, and 87.2% at 32-36 weeks. The majority of PTBs at < 28 weeks were spontaneous (44.2%), followed by PPROM (29.3%) and medically indicated (26.5%). A similar pattern was observed for PTBs at 32-36 weeks, among which 41.5% were spontaneous, 30.7% PPROM, and 27.8% medically indicated. In contrast, PTBs at 28-31 weeks were more frequently medically indicated (40.6%) than PPROM (31.8%) or spontaneous (27.6%). The proportion of PTB was greater for mothers with localized (10.9%) and systemic comorbidities (8.7%), compared to those without comorbidity (4.7%, Table 1). Systemic and localized comorbidities were present in 34.1% and 31.8% of births, respectively (data not in table). Among mothers with systemic comorbidities, medically indicated PTBs (4.1%) were more frequent than PPROM (2.2%) or spontaneous PTBs (2.4%); a similar pattern was observed for mothers with localized comorbidities. Among mothers with no comorbidity, however, spontaneous PTBs (2.4%) were more common (though just as frequent as in mothers with systemic comorbidities). Spontaneous PTBs accounted for the largest proportion (> 41%) of births that were severely (< 28 weeks) and moderately (32-36 weeks) preterm, but medically indicated PTBs (40.6%) accounted for the largest proportion of very PTBs (28-31 weeks).

**Table 1:** Preterm birth (PTB) rates by maternal characteristic, Québec, 1989-2006

	**All PTB %**	**Medically indicated %**	PPROM* %	**Spontaneous^† ^%**	**Total births**
	
**Maternal age**					
< 20 years	8.4	1.9	2.1	4.3	56,647
20-24 years	6.7	1.7	2.0	3.0	260,848
25-29 years	5.8	1.6	1.8	2.4	487,831
30-34 years	5.7	1.8	1.8	2.2	373,906
35-39 years	6.9	2.4	2.2	2.3	130,525
≥ 40 years	8.6	3.5	2.6	2.5	19,979
**Grand multiparity**	6.1	1.7	1.7	2.7	6,010
**Period**					
1989-1993	5.9	1.6	1.7	2.7	454,300
1994-1997	6.3	1.8	1.9	2.6	321,884
1998-2001	6.4	1.9	2.1	2.5	276,252
2002-2006	6.5	2.1	2.1	2.3	277,301
**Comorbidity**					
Systemic	8.7	4.1	2.2	2.4	323,433
Localized	10.9	4.9	2.9	3.0	241,580
No	4.7	0.5	1.7	2.4	839,539
**Total**	6.2	1.8	1.9	2.5	1,329,737

* Preterm premature rupture of membranes

† Spontaneous preterm labour before 37 gestational weeks with intact membranes

PTB was more frequent among mothers with preeclampsia/eclampsia (24.1%), drug dependence (21.4%), and edema/renal disease (17.0%) (Table 2, systemic comorbidity). Among mothers with drug dependence, spontaneous PTB was more frequent (9.8%) than PPROM (6.2%) or medically indicated PTB (5.5%), especially at 32-36 weeks (8.5%). For all other causes of systemic comorbidity, medically indicated PTB was more frequent than PPROM and spontaneous PTB. Gestational diabetes and mental disorders were exceptions as the frequency of PTB was similar across clinical subtypes.

**Table 2:** Preterm birth (PTB) rates according to maternal systemic/localized comorbidity and gestational age (weeks) at delivery, and the proportions of comorbidity among PTBs

	**All PTB %**	**Medically indicated** **%**				PPROM* %				**Spontaneous^†^** **%**				**Comorbidity**^‡ ^**%**	**Total births**
	**< 37**	**< 28**	**28-31**	**32-36**	**< 37**	**< 28**	**28-31**	**32-36**	**< 37**	**< 28**	**28-31**	**32-36**	**< 37**	**< 37**	
	
**Systemic comorbidity**															
Hypertension															
Preeclampsia/eclampsia	24.1	1.0	3.3	16.3	20.6	0	0.1	1.2	1.3	0	0.1	2.1	2.2	8.7	29,915
Pre-existing	11.0	0.2	0.7	5.8	6.7	0.1	0.1	1.9	2.1	0.1	0.1	1.9	2.1	0.8	7,439
Gestational	6.1	0.1	0.2	3.2	3.5	0	0.1	1.1	1.2	0	0	1.3	1.4	1.0	11,380
Cardiovascular disease	10.3	0.2	0.6	4.4	5.3	0.2	0.3	2.1	2.6	0.2	0	2.2	2.5	0.6	4,434
Diabetes															
Pre-existing	13.1	0.1	0.6	5.5	6.1	0.1	0.5	3.1	3.7	0.1	0.3	2.9	3.2	2.7	16,938
Gestational	8.4	0.1	0.2	2.6	2.8	0.1	0.2	2.4	2.7	0	0.1	2.8	2.9	5.1	50,313
Edema/renal disease	17.0	0.6	1.9	9.9	12.4	0.1	0.2	1.6	2.0	0.3	0.1	2.3	2.6	0.8	3,977
Genitourinary infection	8.4	0.2	0.5	2.7	3.3	0.2	0.4	2.0	2.6	0.2	0.1	2.1	2.5	3.2	31,477
General infection	10.0	0.2	0.4	3.2	3.8	0.3	0.4	2.2	2.9	0.3	0.2	2.7	3.2	1.9	16,024
Thyroid disease	8.9	0.2	0.4	3.0	3.7	0.2	0.3	2.3	2.8	0.2	0.2	2.2	2.5	1.4	12,687
Anemia	6.9	0.2	0.4	2.3	2.9	0.2	0.2	1.5	2.0	0.2	0.1	1.7	2.0	11.7	140,410
Drug dependence	21.4	0.2	0.8	4.5	5.5	0.6	0.7	5.0	6.2	0.6	0.7	8.5	9.8	3.9	3,229
Mental disorders	13.2	0.4	0.5	3.7	4.5	0.3	0.6	3.2	4.1	0.4	0.3	3.9	4.6	9.3	7,688
**Localized comorbidity**															
Hemorrhage															
Placental abruption	31.9	1.3	2.4	9.9	13.6	1.3	1.6	3.6	6.6	2.3	1.8	7.6	11.7	8.6	22,278
Placenta previa	35.2	0.8	2.9	26.3	30.0	0.9	0.5	2.4	3.8	0.3	0.2	1.1	1.5	2.3	5,265
Chorioamnionitis	11.0	0.3	0.3	1.4	1.9	1.2	1.2	3.1	5.5	1.0	0.5	2.1	3.6	7.3	55,133
Amniotic sac															
Polyhydramnios	12.5	0.3	0.8	4.9	6.0	0.2	0.3	2.0	2.5	0.3	0.5	3.4	4.1	1.3	8,861
Oligohydramnios	20.6	0.8	1.8	10.4	13.0	1.1	1.4	3.2	5.7	0.3	0.2	1.5	1.9	3.8	15,293
Cervical incompetence	47.4	3.4	1.5	6.4	11.2	6.3	2.7	5.7	14.7	11.3	2.1	8.1	21.5	1.5	2,570
Structural abnormality	13.0	0.6	1.1	5.1	6.7	0.4	0.6	3.2	4.2	0.4	0.2	1.5	2.1	3.1	19,515
Previous cesarean delivery	5.8	0.1	0.2	2.9	3.3	0.1	0.1	1.3	1.5	0.1	0.1	0.9	1.0	8.4	118,608
Fetal anomaly	43.2	8.2	1.9	11.7	21.9	0.2	0.6	2.4	3.2	12	0.6	5.5	18.1	0.5	935
**Total**	6.2	0.1	0.2	1.5	1.8	0.1	0.1	1.7	1.9	0.1	0.1	2.2	2.5		1,329,737

* Preterm premature rupture of membranes

† Spontaneous preterm labour before 37 weeks of gestation with intact membranes

‡ Proportion of comorbidity among PTBs overall

PTB was very common among mothers with major localized co-morbidities, especially cervical incompetence (47.4%), fetal anomalies (43.2%), placenta previa (35.2%) and placental abruption (31.9%) (Table 2, localized comorbidity). For cervical incompetence, spontaneous PTB was more frequent (21.5%) than PPROM (14.7%) or medically indicated PTB (11.2%). For other causes of localized comorbidity, medically indicated PTB was more frequent than PPROM or spontaneous PTB. An exception was chorioamnionitis, for which PPROM was more frequent (5.5%) than medically indicated (1.9%) or spontaneous PTB (3.6%).

Localized comorbidities as a whole were more strongly associated with overall PTB (OR 2.27, 95% CI 2.24-2.31) than systemic comorbidities (OR 1.66, 95% CI 1.63-1.68) (Table 3). Both systemic and localized comorbidities were associated with statistically significant four-fold greater odds of medically indicated PTB. Associations were modest for PPROM but nonetheless statistically significant. In contrast, spontaneous PTB was only associated with localized (but not systemic) comorbidities. Associations were robust to adjustments for covariates.

**Table 3:** Association between maternal systemic/localized comorbidity and preterm birth (PTB) by clinical subtype

	**Unadjusted OR (95% CI)**	Adjusted OR (95% CI)*	**Population attributable fraction**
	
**All PTB**			
Systemic comorbidity	1.67 (1.64-1.69)	1.66 (1.63-1.68)	12.8
Localized comorbidity	2.23 (2.20-2.27)	2.27 (2.24-2.31)	16.9
**Medically indicated PTB**			
Systemic comorbidity	4.11 (4.00-4.22)	4.01 (3.90-4.11)	21.8
Localized comorbidity	4.88 (4.76-5.01)	4.83 (4.71-4.96)	23.3
**PPROM^†^**			
Systemic comorbidity	1.23 (1.19-1.26)	1.18 (1.15-1.22)	8.0
Localized comorbidity	1.85 (1.80-1.90)	1.83 (1.78-1.88)	21.1
**Spontaneous PTB**^‡^			
Systemic comorbidity	0.99 (0.97-1.02)	1.00 (0.97-1.02)	0
Localized comorbidity	1.31 (1.28-1.35)	1.38 (1.34-1.42)	5.8

* Odds ratio (OR), 95% confidence interval (CI) adjusted for maternal age, grand multiparity, and period

† Preterm premature rupture of membranes

‡ Spontaneous preterm labour before 37 weeks of gestation with intact membranes

Almost all categories of systemic and localized comorbidity were associated with greater odds of overall PTB (data not shown). For systemic comorbidities, associations with overall PTB were highest for preeclampsia/eclampsia (OR 5.1, 95% CI 5.0-5.3), drug dependence (OR 3.9, 95% CI 3.5-4.2), and edema/renal disease (OR 3.1, 95% CI 2.8-3.3). For localized comorbidities, odds of overall PTB were highest for cervical incompetence (OR 13.9, 95% CI 12.8-15.0), fetal anomalies (OR 11.8, 95% CI 10.3-13.4), placenta previa (OR 9.4, 95% CI 8.9-10.0), and placental abruption (OR 7.9, 95% CI 7.7-8.2).

Associations between comorbidities and clinical subtype of PTB depended on gestational age (Table 4). For systemic comorbidities, associations with medically indicated PTB were strongest for preeclampsia/eclampsia, chronic hypertension and edema/renal disease, and ORs were largest at 28-31 weeks of gestation. Associations with PPROM also tended to be stronger at 28-31 weeks, especially for mental disorders, genitourinary infection and cardiovascular disease. Hypertension was not associated with greater odds of PPROM at any gestational age (some ORs suggested a protective association). Diabetes was associated with PPROM after 28 weeks (adjusted ORs: 1.3 to 3.5), but the associations were stronger for pre-existing than for gestational diabetes. Drug dependence (adjusted ORs: 3.3 to 5.9), general infection (adjusted ORs: 1.4 to 3.0), and thyroid disease (adjusted ORs: 1.3 to 1.9) were more strongly associated with PPROM at < 32 weeks than 32-36 weeks, while anemia was associated with PPROM at < 32 weeks only (adjusted ORs: 1.7 to 2.1). Associations with spontaneous PTB were strongest for drug dependence and mental disorders at all gestational ages, and pre-existing diabetes at 28-31 weeks. Genitourinary infection was weakly associated with spontaneous PTB at < 28 weeks only (adjusted OR = 1.3), while preeclampsia/eclampsia (but not other categories of hypertension) was weakly associated with spontaneous PTB at 32-36 weeks (adjusted OR = 1.1). Thyroid disease was associated with spontaneous PTB only at 28-31 weeks, while anemia was associated with spontaneous PTB only at < 28 weeks.

**Table 4:** Association between maternal systemic/localized comorbidity and preterm birth according to clinical subtype and gestational age*

	**Medically indicated**			**PPROM^†^**			**Spontaneous**^‡^		
	**< 28 wk**	**28-31 wk**	**32-36 wk**	**< 28 wk**	**28-31 wk**	**32-36 wk**	**< 28 wk**	**28-31 wk**	**32-36 wk**
	
**Systemic comorbidity**									
Hypertension									
Preeclampsia/eclampsia	17.6 (15.4-20.1)	38.7 (35.6-42.1)	18.5 (17.8-19.1)	0.2 (0.1-0.5)	0.6 (0.4-0.9)	0.9 (0.8-1.0)	0.3 (0.2-0.5)	0.7 (0.5-1.1)	1.1 (1.0-1.2)
Pre-existing	3.4 (2.1-5.4)	6.7 (5.1-8.8)	5.1 (4.6-5.6)	1.3 (0.7-2.3)	1.2 (0.7-2.0)	1.1 (0.9-1.3)	1.0 (0.5-1.7)	0.9 (0.5-1.9)	0.9 (0.8-1.1)
Gestational	1.1 (0.5-2.0)	2.1 (1.4-3.1)	2.8 (2.5-3.1)	0.1 (0.0-0.6)	0.5 (0.2-0.9)	0.6 (0.5-0.7)	0.2 (0.1-0.6)	0.1 (0.0-0.5)	0.6 (0.5-0.7)
Cardiovascular disease	2.2 (1.2-4.3)	3.4 (2.3-4.9)	3.0 (2.6-3.4)	1.6 (0.8-3.4)	2.4 (1.5-4.0)	1.3 (1.0-1.6)	1.6 (0.8-2.9)	2.0 (1.1-3.7)	1.1 (0.9-1.3)
Diabetes									
Pre-existing	1.2 (0.8-1.9)	3.2 (2.6-3.9)	3.9 (3.6-4.2)	1.2 (0.8-1.8)	3.5 (2.8-4.4)	2.0 (1.9-2.2)	0.9 (0.6-1.4)	2.2 (1.7-3.1)	1.4 (1.3-1.6)
Gestational	0.6 (0.4-0.8)	1.2 (1.0-1.4)	1.7 (1.6-1.8)	0.5 (0.3-0.7)	1.3 (1.0-1.6)	1.5 (1.4-1.6)	0.3 (0.2-0.4)	0.8 (0.6-1.1)	1.3 (1.3-1.4)
Edema/renal disease	6.8 (4.4-10.3)	11.9 (9.4-14.9)	7.3 (6.6-8.1)	1.4 (0.6-3.3)	1.7 (0.9-3.3)	1.1 (0.8-1.4)	1.8 (1.0-3.4)	0.9 (0.3-2.4)	1.1 (0.9-1.4)
Genitourinary infection	1.7 (1.3-2.3)	2.6 (2.2-3.1)	1.8 (1.7-1.9)	2.1 (1.6-2.7)	2.6 (2.1-3.1)	1.2 (1.1-1.3)	1.3 (1.0-1.7)	1.0 (0.7-1.4)	1.0 (0.9-1.1)
General infection	2.5 (1.8-3.5)	2.2 (1.7-2.8)	2.2 (2.0-2.4)	3.0 (2.3-4.0)	2.8 (2.2-3.6)	1.4 (1.2-1.5)	1.7 (1.3-2.4)	2.1 (1.5-2.9)	1.3 (1.2-1.4)
Thyroid disease	2.3 (1.6-3.4)	2.1 (1.6-2.8)	1.9 (1.7-2.1)	1.9 (1.3-2.9)	1.9 (1.3-2.6)	1.3 (1.2-1.5)	1.1 (0.7-1.7)	1.6 (1.0-2.5)	1.1 (1.0-1.2)
Anemia	2.2 (1.9-2.6)	2.7 (2.4-2.9)	1.6 (1.5-1.7)	2.1 (1.8-2.4)	1.7 (1.5-1.9)	0.9 (0.9-0.9)	1.3 (1.2-1.5)	1.0 (0.8-1.2)	0.7 (0.7-0.8)
Drug dependence	1.8 (0.8-4.4)	5.1 (3.5-7.5)	3.3 (2.8-3.9)	5.9 (3.7-9.3)	4.9 (3.2-7.4)	3.3 (2.8-3.9)	3.9 (2.4-6.2)	6.1 (4.0-9.2)	4.1 (3.6-4.6)
Mental disorders	3.6 (2.5-5.3)	2.6 (1.9-3.6)	2.4 (2.1-2.7)	3.3 (2.2-4.8)	4.1 (3.0-5.5)	1.9 (1.7-2.2)	2.7 (1.9-3.8)	2.6 (1.7-3.9)	1.8 (1.6-2.0)
**Localized comorbidity**									
Hemorrhage									
Placental abruption	27.7 (24.2-31.8)	25.2 (22.9-27.9)	10.7 (10.2-11.3)	24.7 (21.6-28.2)	19.7 (17.5-22.2)	3.0 (2.8-3.2)	30.4 (27.4-33.7)	28.4 (25.3-31.8)	5.1 (4.9-5.4)
Placenta previa	17.7 (12.9-24.2)	30.4 (25.6-36.1)	29.8 (27.9-31.8)	16.8 (12.5-22.7)	6.6 (4.5-9.6)	2.1 (1.8-2.5)	3.4 (2.0-5.9)	3.0 (1.5-5.7)	0.8 (0.6-1.0)
Chorioamnionitis	3.8 (3.3-4.5)	1.5 (1.3-1.8)	0.9 (0.9-1.0)	24.0 (21.5-26.8)	12.4 (11.2-13.6)	2.0 (1.9-2.1)	9.6 (8.7-10.6)	4.5 (3.9-5.2)	1.0 (1.0-1.1)
Amniotic sac									
Polyhydramnios	3.1 (2.0-4.7)	5.1 (4.0-6.5)	3.6 (3.3-4.0)	1.7 (1.0-3.0)	2.4 (1.6-3.5)	1.3 (1.1-1.5)	2.1 (1.4-3.1)	4.1 (3.0-5.6)	1.6 (1.5-1.8)
Oligohydramnios	10.2 (8.4-12.4)	13.0 (11.5-14.8)	8.6 (8.1-9.1)	15.1 (12.8-17.8)	12.6 (10.9-14.6)	2.2 (2.0-2.4)	2.6 (2.0-3.5)	1.7 (1.1-2.5)	0.8 (0.7-0.9)
Cervical incompetence	68.6 (54.6-86.1)	13.6 (9.8-18.9)	7.1 (6.1-8.4)	123 (103-146)	33.4 (26.1-42.7)	6.1 (5.1-7.2)	159 (139-183)	32.4 (24.5-42.9)	6.9 (5.9-7.9)
Structural abnormality	6.8 (5.5-8.3)	6.3 (5.4-7.3)	3.5 (3.2-3.7)	4.2 (3.3-5.2)	4.2 (3.4-5.1)	2.0 (1.9-2.2)	2.5 (2.0-3.2)	2.2 (1.6-2.9)	0.8 (0.7-0.9)
Previous cesarean delivery	1.2 (1.0-1.4)	1.3 (1.1-1.4)	2.0 (2.0-2.1)	1.1 (0.9-1.3)	0.9 (0.7-1.0)	0.8 (0.7-0.8)	0.4 (0.3-0.5)	0.5 (0.4-0.6)	0.4 (0.4-0.4)
Fetal anomaly	159 (123-204)	16.3 (10.2-26.2)	12.4 (10.1-15.3)	3.2 (0.8-12.8)	7.0 (3.1-15.6)	2.3 (1.5-3.5)	142 (115-176)	8.7 (3.9-19.6)	4.1 (3.1-5.5)

* Odds ratio (95% confidence interval) adjusted for maternal age, grand multiparity, and period (rounded to the first decimal to conserve space)

† Preterm premature rupture of membranes

‡ Spontaneous preterm labour before 37 weeks of gestation with intact membranes

Localized comorbidities were generally associated with medically indicated PTB and PPROM at most gestational ages, while the associations with spontaneous PTB tended to be stronger at < 32 weeks of gestation. Previous cesarean delivery was associated with greater odds of medically indicated PTB after 28 weeks, but lower odds of PPROM at 32-36 weeks and spontaneous PTB at all gestational ages.

Attributable fractions suggested that preeclampsia/eclampsia, anemia, placental abruption, structural abnormalities, and chorioamnionitis were important contributors to medically indicated PTB at the population level, though the contribution varied by gestational age (Table 5). Anemia and genitourinary infections, as well as placental abruption, chorioamnionitis, and structural abnormalities, were the largest contributors to PPROM. No particular cause of systemic comorbidity accounted for a substantial fraction of spontaneous PTB, but major localized morbidities including placental abruption, chorioamnionitis, cervical incompetence and structural abnormalities were important contributors, especially at < 32 weeks of gestation.

**Table 5:** Population attributable fractions for maternal systemic/localized comorbidity and preterm birth according to clinical subtype and gestational age*

	**Medically indicated**			PPROM*			**Spontaneous^†^**		
	**< 28 wk**	**28-31 wk**	**32-36 wk**	**< 28 wk**	**28-31 wk**	**32-36 wk**	**< 28 wk**	**28-31 wk**	**32-36 wk**
	
**Systemic comorbidity**									
Hypertension									
Preeclampsia/eclampsia	21.6	38.0	21.9	-1.2	-0.7	-0.2	-1.2	-0.6	0.2
Pre-existing	0.8	1.1	1.6	0	0.1	0.1	0	0	-0.1
Gestational	0.2	1.8	1.8	-4.5	-0.8	-0.4	-2.4	-4.3	-0.3
Cardiovascular disease	0.4	0.8	0.6	0.1	0.5	0.1	0.2	0.3	0.02
Diabetes									
Pre-existing	0.3	2.7	3.4	0.1	3.0	1.2	-0.1	1.4	0.5
Gestational	-1.9	0.6	2.6	-1.5	1.0	1.8	-3.0	-0.8	1.1
Edema/renal disease	1.6	2.9	1.7	0.1	0.2	0.02	0.2	-0.03	0.03
Genitourinary infection	1.7	3.7	1.9	1.7	3.6	0.5	0.7	-0.02	-0.05
General infection	1.8	1.4	1.4	1.6	2.1	0.4	0.9	1.2	0.3
Thyroid disease	1.4	1.0	0.9	0.6	0.8	0.3	0.1	0.5	0.1
Anemia	11.9	14.8	5.9	6.7	6.6	-1.1	3.3	-0.1	-2.9
Drug dependence	0.03	0.9	0.5	0.8	0.9	0.5	0.7	1.1	0.7
Mental disorders	1.7	0.9	0.8	0.9	1.8	0.5	1.0	0.8	0.4
**Localized comorbidity**									
Hemorrhage									
Placental abruption	23.5	21.0	9.8	14.1	17.9	2.4	25.2	22.9	4.5
Placenta previa	3.3	5.9	6.6	2.2	1.2	0.3	0.5	0.4	-0.1
Chorioamnionitis	10.5	2.0	-0.3	31.4	30.7	3.8	25.1	11.7	0.04
Amniotic sac									
Polyhydramnios	1.3	2.3	1.5	0.3	0.8	0.2	0.7	1.8	0.4
Oligohydramnios	8.7	10.3	7.0	8.2	10.2	1.2	1.6	0.6	-0.2
Cervical incompetence	7.2	1.4	0.7	8.1	3.5	0.5	14.6	3.1	0.6
Structural abnormality	41.9	36.0	17.3	14.9	22.9	7.0	11.8	6.2	-1.2
Previous cesarean delivery	1.4	2.3	8.7	0.4	-1.3	-2.3	-5.7	-4.7	-5.4
Fetal anomaly	6.5	0.7	0.5	0.1	0.3	0.1	5.7	0.3	0.1

* Preterm premature rupture of membranes

† Spontaneous preterm labour before 37 gestational weeks with intact membranes

## Discussion

To our knowledge, this study is the first to evaluate relations between systemic and localized maternal comorbidities with PTB by clinical subtype and gestational age. In a large population-based cohort, we demonstrated that comorbidities overall were associated with higher likelihoods of medically indicated PTB, while only comorbidities localized to the reproductive tract were associated with spontaneous PTB. At the population level, several major localized comorbidities (placental abruption, chorioamnionitis, oliogohydramnios, structural abnormality, cervical incompetence) were key contributors to all three clinical subtypes of PTB, especially at < 32 weeks of gestation. In contrast, major systemic comorbidities (preeclampsia, anemia) were the key contributors to medically indicated PTBs, but not spontaneous PTBs. This study is the first to identify anemia as the most important maternal systemic comorbidity contributing to PPROM at the population level.

Hypertension is a major pregnancy complication [26]. We found that preeclampsia/eclampsia was strongly associated with medically indicated PTB, confirming the findings from other studies [1,4,27-30]. Data are conflicting as to whether hypertension may be a stronger determinant of early than of late PTB, but previous studies are limited as they often do not differentiate clinical subtypes of PTB [6,31]. We observed that preeclampsia/eclampsia and pre-existing hypertension were strongly associated with medically indicated PTB at all gestational ages. This was not the case for gestational and unspecified hypertension that were only associated with medically indicated PTB at ≥ 28 weeks, albeit much more weakly. Few studies have evaluated the link between hypertension and spontaneous PTB. Two studies found that pre-existing and gestational hypertension in the US were associated with more than 60% greater odds of spontaneous PTB, but PPROM was not evaluated [29,32]. In contrast, we found that hypertension was not associated with elevated odds of either PPROM or spontaneous PTB. In fact, ORs were protective for some hypertensive disorders. Close monitoring of hypertensive pregnancies may partly explain the protective associations with PPROM and spontaneous PTB, via shifting of potential PTBs to the medically indicated subtype (through early detection and labour induction).

The influence of diabetes, another common pregnancy complication, also depended on clinical subtype of PTB and gestational age. We confirmed that compared to gestational diabetes, pre-existing diabetes was more strongly associated with medically indicated PTB [18,33]. Diabetes was associated with greater odds of PPROM after 28 weeks, but the magnitude was also greater for pre-existing than for gestational diabetes. A strong association with spontaneous PTB at 28-31 weeks was only observed for pre-existing diabetes. Other research also suggests strong associations between pre-existing diabetes and spontaneous PTB [18,29,33]. One study evaluating the odds of very PTB (< 32 weeks) found strong associations with pre-existing type 1 diabetes, but the clinical subtype of PTB was not differentiated [34].

Drug dependence and mental disorders are increasingly recognized as drivers of PTB [35-37]. We observed that drug dependence and mental disorders were associated with all clinical subtypes of PTB at most gestational ages. This finding is novel and important because most studies have focused on infections as a cause of PPROM and spontaneous PTB, while other comorbidities have been less studied [1,8,38]. Psychological or social stress, psychiatric disorders, and substance abuse have been associated with overall PTB [1,5,37,39], while other research has demonstrated associations between depression and medically indicated PTB at < 35 weeks [40], and between anxiety/alcohol use and spontaneous PTB [41]. Our findings confirm an association between infections and all PTB clinical subtypes at all gestational ages [1,8,10,11,32,42]. Prevention of PTB would likely benefit from greater attention to drug dependence and mental disorders given the probable underreporting of these causes in pregnancy (with consequently underestimated attributable fractions) [35,36], since the benefits of treatment for infection are uncertain [7,43,44].

Remaining causes of systemic comorbidity including cardiovascular disease, edema/renal disease, thyroid disease, and anemia were mainly associated with medically indicated PTB at all gestational ages, as observed elsewhere [4,18,30]. Thyroid disease and anemia, however, were mainly associated with PPROM at earlier gestational ages (< 32 weeks). For spontaneous PTB, associations were strong with cardiovascular disease at 28-31 weeks, and weak with thyroid disease at 28-31 weeks and anemia at < 28 weeks. Anemia has been associated with greater odds of spontaneous PTB but not PPROM and medically indicated PTB [45]. Other researchers have observed that thyroid disorders were only associated with PPROM [18], whereas we found that thyroid disorders were also associated with medically indicated and spontaneous PTBs at 28-31 weeks. More research is needed to better understand the relation between these maternal comorbidities and PTB, especially anemia which was associated with large attributable fractions for PPROM and medically indicated PTB.

Localized comorbidities in general were associated with PTB, especially medically indicated PTB and PPROM at most gestational ages. Several studies have found that overall PTB was associated with cervical incompetence, fetal anomalies, placenta previa, placental abruption, and oligohydramnios [1,4,5,17], and fewer have, like ours, demonstrated stronger associations at lower gestational ages for placental abruption, chorioamnionitis, oligohydramnios, cervical incompetence, and fetal factors [5,6,15,46]. Spontaneous PTB has been linked with placenta previa and abruption, while polyhydramnios/oligohydramnios were associated with both PPROM and spontaneous PTB [1,14,42]. Interestingly, we found that previous cesarean delivery was associated with lower odds of PPROM and spontaneous PTB.

This study was subject to limitations. Some comorbidities could not be examined in finer categories due to limited ICD code availability or sample size (e.g., cardiovascular and thyroid disease), and underreporting of certain comorbidities (e.g., drug dependence and mental disorders) is probable. Though hospital data are routinely used for surveillance in Québec [16], validation of diagnostic (or procedure) codes has not been undertaken, and results should be interpreted in light of probable non-differential misclassification which may have attenuated associations. Though spontaneous PTBs that resulted in cesarean deliveries could not be identified and were classified as medically indicated, our results are consistent with other studies indicating greater frequencies of spontaneous PTB compared with other clinical subtypes [1,9,17], which suggests that misclassifications are relatively infrequent. We did not have detailed data on parity or other individual maternal characteristics such as income, race, smoking, genetics, or infertility treatment that are known to be associated with PTB. These characteristics have complex relationships with PTB [1,13,47-53], and may be upstream risk factors influencing PTB at least partly through maternal comorbidities. Generalizability of findings to settings without public health insurance is unclear.

## Conclusions

This study demonstrated that the associations between maternal comorbidities and PTB may differ by clinical subtype and gestational age. Preventive strategies to reduce PTB should target major systemic and localized co-morbidities that account for large proportions of PTBs, especially maternal hypertension, anemia, placental abruption, and structural abnormalities. Better clinical management of these maternal comorbidities may be helpful, but such measures should take into account variation in associations by clinical subtype and gestational age.

## Competing interests

The authors declare that they have no competing interests.

## Authors' contributions

NA and TUNL conceived the study, with contributions from ZCL. NA prepared the data. TUNL/ALP performed the statistical analyses. NA and TUNL interpreted the results with contributions from ALP and ZCL. NA and TUNL reviewed the literature and wrote the manuscript. ALP and ZCL critically revised the manuscript for scientific quality and content. All authors approved the final version for publication.

## Pre-publication history

The pre-publication history for this paper can be accessed here:

http://www.biomedcentral.com/1471-2393/11/67/prepub

## Supplementary Material

Additional file 1 **Definition of systemic and localized maternal comorbidity based on International Classification of Diseases (ICD)-9 codes**. International Classification of Disease codes (ninth revision) for the causes of maternal comorbidity analyzed in the current study. Click here for file
