# Supplementary material for: Association between maternal comorbidity and preterm birth by severity and clinical subtype: retrospective cohort study
Source: BMC Pregnancy Childbirth. 2011 Oct 4;11:67. doi: 10.1186/1471-2393-11-67 (PMC3206460; doi:10.1186/1471-2393-11-67)
Supplement: Additional file 1 — Definition of systemic and localized maternal comorbidity based on International Classification of Diseases (ICD)-9 codes. International Classification of Disease codes (ninth revision) for the causes of maternal comorbidity analyzed in the current study. [file 1471-2393-11-67-S1.DOC]

**Additional file 1 – Definition of systemic and localized maternal comorbidity based on International Classification of Diseases (ICD)-9 codes**

|  | **ICD-9** |
| --- | --- |
| **Systemic comorbidity** |  |
| Hypertension* |  |
| Preeclampsia/eclampsia | 642.4-642.7 |
| Pre-existing | 642.0-642.2 |
| Gestational | 642.3 |
| Unspecified | 642.9 |
| Cardiovascular disease | 648.5, 648.6 |
| Diabetes* |  |
| Pre-existing | 648.0 |
| Gestational | 648.8 |
| Edema/renal disease | 646.1, 646.2 |
| Genitourinary infection | 646.6 |
| General infection | 647 |
| Thyroid disease | 648.1 |
| Anemia | 648.2 |
| Drug dependence | 648.3 |
| Mental disorder | 648.4 |
| Other | 643, 646.3, 646.4, 646.7-646.9, 648.7, 648.9 |
| **Localized comorbidity** |  |
| Hemorrhage* |  |
| Placental abruption | 641.2 |
| Placenta previa | 641.0, 641.1 |
| Other | 641.3, 641.8, 641.9 |
| Chorioamnionitis | 658.4 |
| Amniotic sac* |  |
| Polyhydramnios | 657 |
| Oligohydramnios | 658.0 |
| Unspecified | 658.8, 658.9 |
| Cervical incompetence | 654.5 |
| Structural abnormality (uterus/cervix/vagina/vulva) | 654.0, 654.1, 654.3, 654.4, 654.6-654.9 |
| Previous cesarean delivery | 654.2 |
| Fetal factors* |  |
| Anomaly (central nervous system  malformation/chromosomal abnormality) | 655.0, 655.1 |
| Other fetal factor | 655.2-655.9 |

* For cases with more than one documented ICD code, priority for categorization was given in descending order among the sub-categories (from top to bottom). Note that this did not apply to comorbidities that were coded yes/no.
